# Inhibition of the alpha-ν integrins with a cyclic RGD peptide impairs angiogenesis, growth and metastasis of solid tumours *in vivo*

**DOI:** 10.1038/sj.bjc.6600141

**Published:** 2002-03-04

**Authors:** M A Buerkle, S A Pahernik, A Sutter, A Jonczyk, K Messmer, M Dellian

**Affiliations:** Institute for Surgical Research, Klinikum Grosshadern, Marchioninistrasse 15, Ludwig-Maximilians-University, 81377 Munich, Germany; Merck KGaA Preclinical Research, Frankfurter Strasse 250, D-6427 Darmstadt, Germany; Department of Otorhinolaryngology, Klinikum Grosshadern, Marchioninistrasse 15, Ludwig-Maximilians-University, 81377 Munich, Germany

**Keywords:** angiogenesis, tumour, antiangiogenesis, α_v_-integrins, RGD-peptides

## Abstract

Anti-angiogenetic cancer therapy is a potential new form for treatment of solid tumours. The α_v_-integrins (α_v_β_3_, α_v_β_5_) mediate the contact of activated endothelial cells to proteins of the extracellular matrix during tumour angiogenesis as a prerequisite for survival of endothelial cells. The aim of this study was to investigate the effects of application of a methylated cyclic RGD-peptide as an α_v_-integrin antagonist on angiogenesis, microcirculation, growth and metastasis formation of a solid tumour *in vivo*. Experiments were performed in the dorsal skinfold preparation of Syrian Golden hamsters bearing the amelanotic hamster melanoma A-Mel-3. Animals were injected intraperitoneally with a methylated cyclic RGD-peptide every 12 h, the control group received an inactive peptide. Microcirculatory parameters of tumour angiogenesis including functional vessel density, red blood cell velocity, vessel diameter and leucocyte–endothelium interaction were analysed using intravital microscopy. In an additional study the effects on growth and metastasis of subcutaneous A-Mel-3 were quantified. Functional vessel density was markedly reduced on day 3 in treated animals compared to controls (37.2±12.1 *vs* 105.2±11.2 cm^−1^; mean±s.e.m.; *P*<0.05) and increased subsequently in both groups. Red blood cell velocity at day 3 was below values of controls (0.026±0.01 *vs* 0.12±0.03 mm s^−1^; *P*<0.05). No differences were observed in vessel diameters and leucocyte–endothelium interaction was almost absent in both groups. Furthermore, growth and metastasis of subcutaneous tumours after administration of the cyclic RGD-peptide was significantly delayed in comparison to controls (*P*<0.05). Inhibition of α_v_-integrins by a cyclic RGD-peptide resulted in significant reduction of functional vessel density, retardation of tumour growth and metastasis *in vivo*. Taken together, these results implicate RGD-peptides as agents which have anti-tumour and anti-metastatic activity *in vivo*.

*British Journal of Cancer* (2002) **86**, 788–795. DOI: 10.1038/sj/bjc/6600141 www.bjcancer.com

© 2002 Cancer Research UK

## 

Angiogenesis, the growth of new blood vessels from pre-existing vessels, is involved in several physiological and pathological situations like wound healing (Clark *et al*, 1982) and inflammatory diseases (Auerbach and Auerbach, 1994; Folkman, 1995). For growth and metastasis of solid tumours angiogenesis is the key prerequisite (Folkman, 1990, 1995). Furthermore, the extent of vascularisation in solid tumours is an independent prognostic factor for survival and therapeutic outcome (Weidner *et al*, 1991, 1993; Graham *et al*, 1994). Recently, the concept of anti-angiogenetic therapy for tumours has been proven in animals (O'Reilly *et al*, 1997), and this new therapeutic strategy will most likely gain clinical importance. However, development of anti-angiogenetic drugs based on the understanding of the mechanisms of angiogenesis is still in the early stages.

There is emerging evidence that tumour angiogenesis is regulated by the balance of pro-angiogenic and anti-angiogenic factors (Liotta *et al*, 1991; Hanahan and Folkman, 1996). To establish a vascular system in the tumour it requires the cooperation of a variety of molecules that regulate cellular processes such as activation of endothelial cells, proliferation, modulation of the extracellular matrix (ECM), invasion, migration and vascular remodelling. Members of the integrin class of cell adhesion receptors play a key role in cell–cell and cell–ECM interaction during the growth of new blood vessels (Hynes, 1992; Luscinskas and Lawler, 1994). Especially the α_v_-integrins, combined with distinct β subunits, exhibit a high expression in angiogenesis (Cheresh, 1992; Eliceiri and Cheresh, 1999). α_v_-integrins on endothelial cells are capable of recognising a variety of ECM proteins with an exposed Arg-Gly-Asp (RGD) sequence, including vitronectin, fibronectin, fibrinogen, thrombospondin, proteolysed collagen, von Willebrand factor and osteopontin (Brooks, 1996). Consequently, antibodies or synthetic RGD-containing peptides directed against the α_v_-integrins inhibited *in vitro* endothelial tube formation or microvessel outgrowth in previous experiments (Nicosia and Bonanno, 1991; Brooks, 1996; Bayless *et al*, 2000). Most recently it was shown that the expression of α_v_β_3_ and α_v_β_5_ by the microvascular endothelium of neuroblastoma is associated with the aggressiveness of these tumours (Erdreich-Epstein *et al*, 2000).

The importance of α_v_-integrins in tumour angiogenesis was especially elucidated by Cheresh and colleagues in a series of experiments (Brooks *et al*, 1994a,b). An anti-α_v_β-mAb limited blood vessel growth of human melanomas, implanted in the chorioallantoic membrane (Brooks *et al*, 1994b). Application of cyclic RGD peptide antagonists for α_v_β_3_ and α_v_β_5_ disrupted angiogenesis on the CAM and lead to regression of transplanted tumours (Brooks *et al*, 1994a). Interestingly, the antagonists did not affect pre-existing vessels. The mechanism of action of α_v_-antagonists in blocking angiogenesis seems to be related to their ability to selectively promote apoptosis of endothelial cells in newly sprouting blood vessels (Brooks *et al*, 1994b; Stromblad *et al*, 1996; Eliceiri and Cheresh, 1999) and by affecting the function of proteolytic enzymes such as metalloproteinases (Brooks *et al*, 1996; 1998).

Recently, a methylated cyclic RGD peptide has been synthesised as highly active and selective ligand for the α_v_-integrin receptor (Dechantsreiter *et al*, 1999). The effects of anti-angiogenic treatment using this cyclic RGD-peptide on growth and angiogenesis of solid tumours *in vivo* have not been studied so far. The dorsal skinfold chamber preparation, bearing a solid homologous transplanted tumour, represents a unique and well established tool to study tumour angiogenesis non-invasively and continuously over a prolonged period of time *in vivo* (Asaishi *et al*, 1981; Endrich *et al*, 1982). Direct observation of tumour microvessels in the dorsal skinfold chamber preparation may provide further insights into the alterations of angiogenesis during the application of a potential angiogenesis inhibitor. It was therefore the aim of the present study to investigate the effects of the cyclic RGD peptide on tumour angiogenesis, microcirculation, growth and metastasis of solid tumours *in vivo*.

## MATERIALS AND METHODS

Experiments were performed with male Syrian Golden Hamsters (6–8 weeks old, 50–60 g body weight (bw)) in accordance with institutional guidelines after approval of the animal committee of the Bavarian state. The animals were housed one per cage and had free access to tap water and standard laboratory food throughout the experiments. Animals were inspected at least two times a day by specialised animal colony staff to assure a normal clinical condition (including appearance, posture, behaviour and physiological responses). Particular attention was given to body weight and any signs of discomfort, ulceration or inflammation. All surgical procedures were performed under pentobarbital anaesthesia (50 mg kg^−1^ b.w., i.p.; Nembutal, Sanofia-Leva, Hannover, Germany). The experiments met all the standards required by the UKCCCR guidelines for the welfare of animals in experimental neoplasia (United Kingdom Co-ordinating Committee on Cancer Research (UKCCCR), 1998).

### Dorsal skinfold chamber preparation

For quantification of tumour angiogenesis, a dorsal skinfold chamber preparation consisting of two symmetrical titanium frames was surgically implanted into the dorsal skin as described earlier in detail (Asaishi *et al*, 1981; Endrich *et al*, 1982). This is considered to cause the least distress upon the animal because the dorsal skin is extremely expansible. Following implantation of the transparent chamber and a recovery period of 48 h from anaesthesia and microsurgery, preparations fulfilling the criteria of an intact microcirculation were implanted with 2×10^5^ cells of the amelanotic melanoma of the hamster A-Mel-3 (Fortner *et al*, 1961). Fine polyethylene catheters (PE10, inner diameter 0.28 mm) were permanently inserted into the right jugular vein 48 h before first measurement to allow injection of fluorescent agents for intravital fluorescence microscopy. The animals tolerated the dorsal skinfold chamber well and showed no signs of discomfort.

### Assessment of tumour angiogenesis and growth by intravital microscopy

Quantification of tumour angiogenesis using intravital fluorescence microscopy have been described in detail elsewhere (Asaishi *et al*, 1981; Dellian *et al*, 1995, 1996). In brief, the awake animal was trained to crawl into a transparent plastic tube and was placed on a custom-made stage under a modified Leitz microscope (Orthoplan; Leitz, Munich, Germany). FITC-labelled dextran (Sigma, Deisenhofen, Germany; MW 500000; 0.05–0.1 ml of a 5% solution in 0.9% NaCl) as a plasma marker and rhodamine 6G (Molecular Probes, Eugene, OR, USA; 0.04 ml of a 0.05% solution in 0.9% NaCl) to label white blood cells *in vivo*, were injected i.v. to visualise microcirculation and leucocyte–endothelium interaction, respectively. The used dosage of the applied fluorescence dye does not have any toxic effect upon the animal. Selective observation of FITC-labelled plasma was possible using epi-illumination with a 100 W mercury lamp attached to a Ploemopack illuminator with a Leitz I2/3 filter block (excitation 450–490 nm, emission ⩽515 nm) and rhodamine 6G-stained leucocytes were visualised using a Leitz N2 filter block (excitation 530–560 nm, emission ⩽580 nm). Intravital microscopy was performed 3, 5, 9 and 13 days after implantation of the tumour cells. At least 3–5 sites of interest per animal were randomly selected in centre and periphery of the tumour. Images were acquired by a SIT video camera (C2400-08; Hamamatsu Herrsching, Germany) and recorded on S-VHS video tape (Sony) for subsequent analysis. Parallel to microvascular observations the area covered by tumour in the chamber preparation was registered on videotape using a Leitz macroscope with video camera (XC-77; Sony) to evaluate tumour growth. The tumour area (mm^2^) was quantified from videotape by digital image analysis.

Analysis of microcirculatory parameters was performed off-line from video tape by an image analysis system (Cap Image; Zeintl, Heidelberg, Germany). This system described in detail by Zeintl *et al* (1989) and Klyscz *et al* (1997) allows measurement of functional vessel density (FVD) as a parameter of angiogenic activity (Dellian *et al*, 1996). FVD is defined as the total length of perfused microvessels per unit area of observation and is given in cm^−1^. Red blood cell velocity (vRBC) was quantified with the line shift diagram method according to Klyscz *et al* (1997) in mm s^−1^. Rolling leucocytes were defined as population of cells temporarily interacting with the vessel wall and thus having a velocity at least 50% below vRBC in the same vessel. Adherent leucocytes were given as the number of leucocytes remaining stationary for at least 30 s per square millimetre of vessel wall surface (Atherton and Born, 1972; Dellian *et al*, 1996).

### Evaluation of tumour growth and metastasis

Male Syrian Golden Hamsters (weight, age and housing as described above) were anaesthetised with pentobarbital and the dorsal skin was shaven and chemically depilated (Pilcamed, Schwarzkopf, Germany). Cells (4 to 6×10^6^) of the A-Mel-3 were suspended in a 10 μl volume and injected s.c. over the lumbosacral region of the dorsal skin. This is considered to cause the least distress upon the animal because the dorsal skin is extremely expansible. Starting on day 5 after tumour cell implantation, the longer (l) and shorter (w) perpendicular axes and the height (h) of each tumour nodule were measured with callipers every other day. Metastases of the animals were determined by palpation of axillar and inguinal lymph nodes. The day when metastases were first palpable the animal was defined as metastasised. To minimise the pain and distress of the animals no biopsies were taken from the lymph nodes during the observation time. This was due to the known fact that the axillar or inguinal lymph nodes were the most frequent sites of metastasis formation (Fortner *et al*, 1961; Weiss *et al*, 1990). After the end of the experiments when animals were euthanised, biopsies were taken to confirm histologically the invasion of the lymph nodes. Tumour volume was calculated according to the formula V_t_=0.837×l×w×h (Weiss *et al*, 1990). Animals were observed for 15 to 17 days until tumours reached a maximum volume of 7 cm. The A-Mel-3 tumour does not have severe side effects during this early period of growth.

### Treatment and experimental groups

The cyclic integrin α_v_ antagonist EMD121974 (cyclic Arg-Gly-Asp-D-Phe(N-methyl)Val) (Dechantsreiter *et al*, 1999) and the control peptide EMD135981 (cyclic Arg-β-Ala-Asp-D-Phe(N-methyl)Val) were synthesised and characterised at Merck KgAa (Darmstadt, Germany). In pilot studies pharmacokinetics of the peptides were compared after intravenous and intraperitoneal injection in hamsters. Plasma half-life time of the cyclic RGD-peptide after intraperitoneal injection was 47 min, and the area under the curve was similar after intraperitoneal and intravenous administration. A subsequent pilot study demonstrated a slightly higher effect on tumour growth after administration of 30 mg kg^−1^ every 12 h compared to 30 mg every 2 days. Therefore, we chose the following set-up for administration: Peptides were dissolved in PBS (10 mg ml^−1^). One day after implantation of tumour cells, animals were randomly assigned to two groups (*n*=6) and RGD-peptide EMD121974 or control-peptide EMD135981 were given i.p. every 12 h (30 mg kg^−1^ b.w.) until the end of the experiments.

### Statistical analysis

Results are presented as mean±s.e.m. Data were evaluated using Wilcoxon–Mann–Whitney *U* and Kruskal–Wallis test, respectively (SigmaStat; Jandel Scientific, San Rafael, CA, USA). Metastasis analysis was performed according to the Kaplan–Meier method (Kaplan and Meier, 1958) and the differences were compared for statistical significance using the Cox-*F* test (Lee, 1975, 1980) with the statistical software program Statistica (StatSoft, Inc, 1997, Tulsa, OK, USA). *P* values smaller than 5% were considered to be significant.

## RESULTS

### Functional vascular density

**Figure 1:**
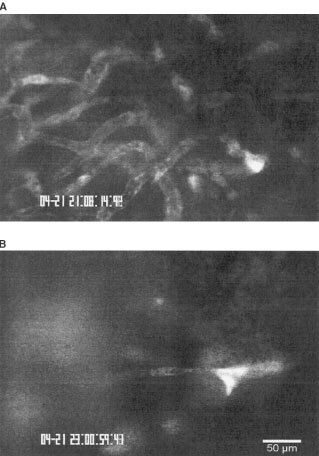
Images of tumour microcirculation 3 days after tumour cell implantation acquired by intravital microscopy after injection of the plasma marker FITC-Dextran. (**A**) Tumour vasculature of animals treated with the control peptide EMD135981 already appears as dense, chaotic structured network. (**B**) In the RGD peptide treated group (EMD121974) only individual sprouts as signs of early angiogenesis were visible. Bar, 50 μm.

**Figure 2:**
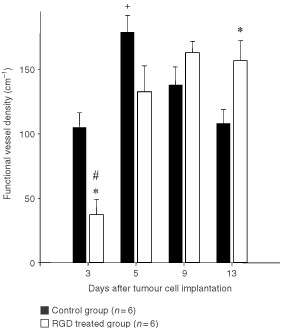
Functional vessel density (mean±s.e.m.) of control group and RGD treated group. Measurements were performed 3, 5, 9 and 13 days after tumour cell implantation by intravital microscopy. Animals were treated every 12 h (30 mg kg^−1^) beginning at day 1 after tumour cell implantation until day 13. *P<0.05 *vs* corresponding controls; #*P*<0.05 *vs* day 9 and 13; ^+^*P*<0.05 *vs* day 3 and 13.

Three days after tumour cell implantation intravital microscopy already revealed an early vascular network in controls (Figure 1

). Short, thin-walled vessels building anastomosis and loops were characteristic for tumour vasculature at this day in the control group (Figure 1A). Intravital microscopy elicited early angiogenesis on day 3 in the RGD treated group. Sometimes vascular sprouts were visible without red cell perfusion (Figure 1B). Quantitative analysis confirmed these observations (Figure 2

). Functional vessel density was significantly reduced on day 3 in treated animals in comparison to controls (32.7±12.1 *vs* 105.2±11.2 cm^−1^; *P*<0.05). Already on day 5, FVD of the control group reached the maximum value and decreased slightly thereafter until the end of the experiments (day 13). In the treated group the highest value of FVD was observed not before day 9, and stayed nearly at the same level afterwards. On day 13 after tumour implantation, FVD of controls was significantly below values of treated animals (108.2±10.6 *vs* 156.9±15.6 cm^−1^; *P*<0.05).

### RBC velocity, vessel diameter and leucocyte–endothelium interaction

**Figure 3:**
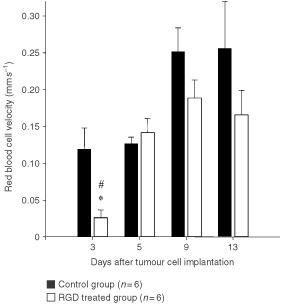
Red blood cell velocity (mean±s.e.m.) of control group and RGD treated group. *P<0.05 *vs* corresponding controls; #*P*<0.05 *vs* treatment day 9 and day 13.

**Figure 4:**
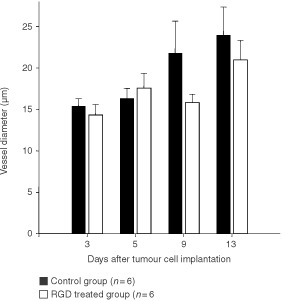
Vessel diameters (mean±s.e.m.) as measured by intravital microscopy of control group and RGD treated group.

Similar to vessel density, RBC velocity (vRBC) in tumour vessels of treated animals was markedly below values of controls on day 3 (0.026±0.01 *vs* 0.12±0.03 mm s^−1^; *P*<0.05; Figure 3

). On days 9 and 13, vRBC was slightly lower in treated animals than in animals receiving the control peptide. Analysis of vessel diameters revealed no differences between animals treated with RGD peptide or control peptide (Figure 4

). Leucocyte–endothelium interaction was almost absent in tumour microvessels of both groups.

### Tumour growth in the dorsal skinfold chamber

**Figure 5:**
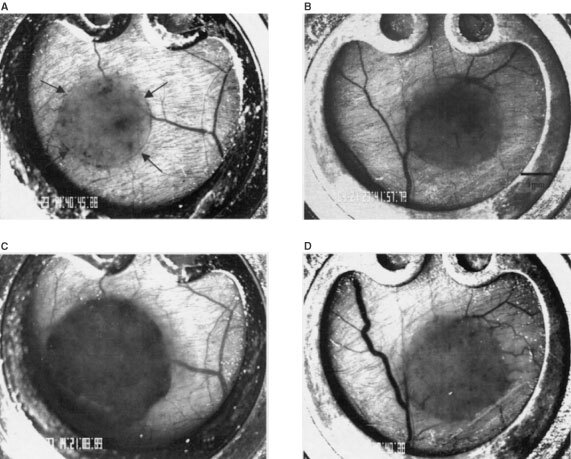
Images of the A-Mel-3 tumour in the dorsal skinfold chamber acquired by video macroscopy. (**A**) Tumour of control group 5 days after tumour cell implantation. Arrows define the tumour margin. (**C**) The same tumour 4 days later. (**B**) Corresponding tumour of RGD treated group on day 5 and (**D**) same tumour on day 9 after tumour cell implantation. Bar 1 mm.

**Figure 6:**
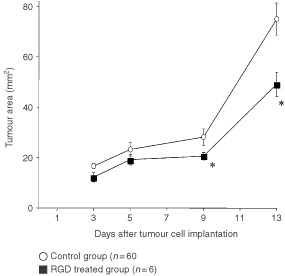
Area of the A-Mel-3 tumour of the control group and the RGD treated group in the dorsal skinfold chamber measured on day 3, 5, 9 and 13 after tumour cell implantation (mean±s.e.m.). *P<0.05 *vs* corresponding controls.

In early tumour growth on day 5 the size of treated tumours was similar to controls (Figure 5A,B

). After establishment of tumour microcirculation, rapid growth of the malignant tissue was possible (Figure 5C). In contrast, growth of the RGD treated tumour was delayed (Figure 5D). Quantitative data confirmed that treated tumours were slightly smaller than controls on days 3 and 5, tumour area was significantly reduced on days 9 (20.5±1.4 *vs* 28.1±3.2 mm^2^; *P*<0.05) and 13 (49.1±4.8 *vs* 80.94±6.1 mm^2^; *P*<0.05; Figure 6

). Due to continuous growth, the tumours obscured the preparations in the control group after 13 days.

### Subcutaneous tumour growth and metastasis

**Figure 7:**
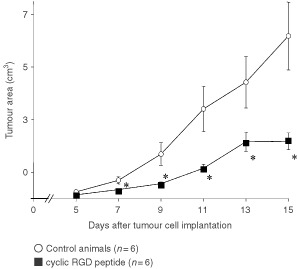
Tumour growth curves of animals with subcutaneously implanted solid A-Mel-3 tumours. Changes in tumour volume are presented for control animals and following application of cyclic RGD peptide. Values are indicated as mean±s.e.m. *P<0.05 *vs* controls.

**Figure 8:**
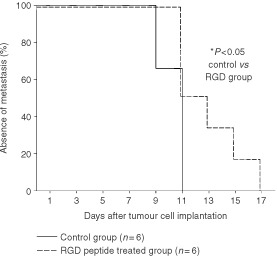
Metastasis formation presented as a Kaplan–Meier curve. Animals of the control group and animals of the RGD peptide treated group were examined for axillar and inguinal lymph node metastases during evaluation of tumour growth. Metastasis formation was significantly delayed following therapy with the RGD peptide. *P<0.05 *vs* controls.

Quantitative data demonstrated that A-Mel-3 tumours treated with the control peptide EMD135981 showed nearly exponential growth (Figure 7

). Administration of the α_v_-antagonist EMD121974 delayed tumour growth for an average of 3.5±0.7 days in comparison to controls: Volume of the treated tumours was significantly smaller in comparison to controls on days 7 until 15. As shown in the Kaplan–Meier curve (Figure 8

) lymph node metastasis were first palpable in two animals of the control group on day 9. Two days later palpable metastasis was found in all control animals. In contrast, 50% of the animals treated with RGD peptide were free of metastasis on day 11. It lasted a total of 17 days, 6 days longer than in controls, until the last animal of the anti-angiogenic treated group showed palpable metastasis of this aggressive, very rapidly growing tumour. At the end of the observations when tumour size reached a maximum volume of 7 cm, animals were put to sleep with a high dose of pentobarbital. Metastasis invasion was histologically confirmed from lymph node biopsies.

## DISCUSSION

α_v_-integrins are receptors for a large number of molecules with an exposed RGD sequence. They are involved in many cell–matrix recognition and cell–adhesion phenomena (Hynes, 1992; Luscinskas and Lawler, 1994). Recent observations revealed that they play an important role in tumour angiogenesis and metastasis (Brooks *et al*, 1994b; Marshall and Hart, 1996). Cyclic RGD peptides have been developed as selective inhibitors for the α_v_-integrins (Gurrath *et al*, 1992; Dechantsreiter *et al*, 1999). The present study was based on earlier observations showing a potent inhibitory effect on tumour angiogenesis using RGD peptides as α_v_-Integrin antagonists (Eliceiri and Cheresh, 1999). Our results have shown that treatment with the methylated cyclic RGD-peptide resulted in significant reduction of functional vessel density, associated with retardation of tumour growth and metastasis formation.

### Animal model

It is well known that caution must be exercised in the extrapolation of *in vitro* observations to the *in vivo* situation, specially concerning experiments about tumour angiogenesis (Jain *et al*, 1997). The present tumour model (Fortner *et al*, 1961; Asaishi *et al*, 1981; Endrich *et al*, 1982) was chosen because detailed information and experience is available about microcirculatory and morphologic changes of the amelanotic melanoma A-Mel-3 during tumour angiogenesis and growth. A broad spectrum of knowledge could be gathered for more than 20 years concerning the tumour profile, including features like growth rate, invasion of organ systems, and sensitivity to various therapeutic regimens. The A-Mel-3 is an early metastasising, exponentially growing aggressive tumour which does not show spontaneous remission. It is therefore suitable for testing the effects of therapeutic modalities. Use of the dorsal skinfold chamber preparation of a solid tumour combined with intravital microscopy and subsequent quantitative microvascular analysis provides an excellent and well established tool for repetitive, direct evaluation of tumour angiogenesis and microcirculation during a long period of anti-angiogenic treatment (Borgstroem *et al*, 1996; Dellian *et al*, 1996; Jain *et al*, 1997; Vajkoczy *et al*, 2000).

### Effects of treatment with RGD-peptides on subcutaneous tumour growth and metastasis

The growth and metastatic properties of solid tumours are directly influenced by the process of angiogenesis (Folkman, 1990). In early tumour growth, nutrition of tumour cells is guaranteed by diffusion from the environment. Newly formed blood vessels provide the basis for nutrition and spreading of tumour cells, one of the most fearsome aspects of cancer (Fidler and Ellis, 1994). In our experiments subcutaneous tumour growth was nearly exponential in control animals. Treatment with the RGD peptide integrin antagonist resulted in significant delay of tumour growth until the end of the experiments. Furthermore, metastasis formation was inhibited: At the time when lymphatic metastasis was palpable in all control animals, only 50% of treated animals showed metastasis. It is well known that the risk of metastasis increases with the number and density of tumour vessels (Weidner *et al*, 1991, 1993) and that growth of metastasis may be angiogenesis dependent as well (Fidler and Ellis, 1994; Saaristo *et al*, 2000). Furthermore, growth of metastasis and the primary tumour may be connected: Folkman and coworkers demonstrated that some primary tumours inhibit the growth of their metastases by endogenous anti-angiogenic mediators that are released by the primary tumour (O'Reilly *et al*, 1994). Sckell *et al* (1998) have shown evidence that anti-angiogenic effects observed at a secondary site correlate with the tumour burden at the primary site. In our study metastases became earliest palpable on day 9 after tumour cell implantation in controls and approximately 50% of the RGD-treated animals showed metastases with a tumour size comparable to the metastatic control tumour. Former studies with surgical excision of the A-Mel-3 have demonstrated that this fast growing, well perfused tumour metastasises already prior to day 5 after implantation of tumour cells (Gamarra *et al*, 1993). Inguinal or axillary metastases become regularly palpable between days 9 and 11 after tumour cell implantation in untreated animals or following local tumour therapy (Dellian *et al*, 1994). Thus, appearance and growth of metastasis in our tumour model is very closely related to the time after implantation of tumour cells, but less to the tumour burden of the animals. Our data from intravital microscopy revealed already on day 3 an early vascular network in controls making an early spreading of tumour cells possible, whereas the reduced vessel density in the RGD-treated group may decrease the probability of tumour cell spreading. Exact mechanisms of metastasis inhibition by anti-α_v_ integrin-treatment in this study, however, are unknown. Reduced metastasis formation may be a consequence of (a) inhibition of angiogenesis at the primary tumour site and thus delayed spreading of tumour cells from the primary tumour and (b) inhibition of angiogenesis in the tumour metastasis, resulting in a delay of growth of tumour metastasis.

Based on previous pharmakokinetic studies, anti-angiogenic therapy was performed by intraperitoneal injections of 30 mg kg^−1^ of the cyclic RGD-peptide twice a day. Plasma half-life time of the cyclic RGD-peptide after intraperitoneal injection was 47 min. However, these data allowed no conclusion on the duration of the peptides' binding to α_v_-integrins, which may not last the 12 h until next administration. Remarkably, no side effects were observed in treated animals indicating low toxicity of the therapeutic regiment: Body weight increased similar in control and treated animals, and animals showed normal behaviour without signs of any discomfort.

### Effects of treatment with RGD-peptides on functional vessel density and tumour microcirculation

With our second series of experiments we wanted to address the question whether the inhibitory effect on the subcutaneous tumour growth and metastasis may be attributed to changes in tumour angiogenesis and microcirculation. By intravital microscopy, the activity of angiogenesis in tumour tissue can be most accurately quantified by measurement of functional vessel density (FVD), i.e. the total length of perfused microvessels per unit area of observation (Borgstroem *et al*, 1996; Dellian *et al*, 1996; Jain *et al*, 1997; Vajkoczy *et al*, 2000).

Comparable to former observations by Endrich *et al* (1982), FVD of the control group reached a maximum value at day 5 after tumour cell implantation, and decreased thereafter whereas tumours continued to grow. This ‘physiological’ decline of tumour vessel density in the later growth phases is probably related to malnutrition and onset of necrosis in the tumour centre: Tumour blood vessels are more leaky than are vessels of normal tissues (Gerlowski and Jain, 1986). The increased fluid extravasation leads to an elevated interstitial fluid pressure (IFP) and low perfusion pressure in tumour vessels, mainly in the centre of the tumour (Peters *et al*, 1980; Wiig *et al*, 1982). This finally results in hypoxia and necrosis formation of central parts of the melanoma, whereas the tumour margin is still sufficiently supplied with blood (Netti *et al*, 1999). On that score the data of the RGD treated group showed three remarkable distinctions: (a) the FVD was significantly lower in early angiogenesis; (b) reached the maximum value not before day 9; and (c) thereafter stayed nearly at the same level until the end of the experiments. Vessel growth was especially inhibited and delayed in the early stage of tumour growth on day 3, whereas differences in tumour growth became first visible later, beginning on day 9 after tumour implantation in the dorsal skinfold chamber, presumably as a consequence of inhibition of angiogenesis. The lack of ‘physiological’ decline of tumour vessel density observed following anti-α_v_ integrin-treatment may be related to continuous delay of tumour growth.

Analysis of red blood velocity during tumour angiogenesis revealed differences only at day 3 following tumour implantation. At this time point, control tumours already exhibited a dense vascular network, whereas in tumours following anti-angiogenic treatment few sprouts as signs of early angiogenesis were visible, which contained erythrocytes, but often no movement of red blood cells was seen yet. In contrast to results of studies of the effects of inhibition of VEGF on tumour microvasculature (Yuan *et al*, 1995; Borgstroem *et al*, 1996), vessel diameters were similar in control and treated tumours of the present study and increased at best slightly during tumour growth. These differences are most likely related to the divergent targets of anti-angiogenic therapeutic regimens. Although leucocyte–adhesion is discussed to be relevant for angiogenesis (Folkman and Shing, 1992; Ferrara, 1996), leucocyte–endothelium interaction was almost absent in both groups. Thus the known reduced expression of leucocyte adhesion molecules in tumour endothelium (Dellian *et al*, 1995; Borgstroem *et al*, 1997) seems to be not affected by anti-angiogenic therapy directed against α_v_-integrins.

## CONCLUSIONS

Inhibition of α_v_-integrins by a cyclic RGD-peptide resulted in significant reduction of functional vessel density, retardation of tumour growth and metastasis *in vivo*. The anti-tumor efficacy was associated with remarkable low toxicity of this compound. Administration and dosage of the cyclic RGD-peptide may be further optimised in future studies. Currently, clinical studies are performed to test safety of anti-angiogenic therapy with this cyclic RGD-peptide in humans.
